# Mesenchymal stem cells inhibit T-cell function through conserved induction of cellular stress

**DOI:** 10.1371/journal.pone.0213170

**Published:** 2019-03-14

**Authors:** Adam G. Laing, Giorgia Fanelli, Andrei Ramirez-Valdez, Robert I. Lechler, Giovanna Lombardi, Paul T. Sharpe

**Affiliations:** 1 MRC Centre for Transplantation, School of Immunology and Microbial Sciences, King's College London, United Kingdom; 2 Centre for Craniofacial and Regenerative Biology, King’s College London, United Kingdom; University of Miami School of Medicine, UNITED STATES

## Abstract

The physiological role of mesenchymal stem cells (MSCs) is to provide a source of cells to replace mesenchymal-derivatives in stromal tissues with high cell turnover or following stromal tissue damage to elicit repair. Human MSCs have been shown to suppress *in vitro* T-cell responses via a number of mechanisms including indoleamine 2,3-dioxygenase (IDO). This immunomodulatory capacity is likely to be related to their *in vivo* function in tissue repair where local, transient suppression of immune responses would benefit differentiation. Further understanding of the impact of locally modulated immune responses by MSCs is hampered by evidence that IDO is not produced or utilized by mouse MSCs. In this study, we demonstrate that IDO-mediated tryptophan starvation triggered by human MSCs inhibits T-cell activation and proliferation through induction of cellular stress. Significantly, we show that despite utilizing different means, immunomodulation of murine T-cells also involves cellular stress and thus is a common strategy of immunoregulation conserved between mouse and humans.

## Introduction

Mesenchymal stem cells (MSCs) is the generic name given to tissue-resident adult stromal stem cells that are capable of differentiating into a number of mesodermal lineages *in vitro* [1]. In addition to their ‘stem cell properties’, MSCs have been shown to exhibit broad and potent immunomodulatory effects *in vitro* and *in vivo* [2–7]. As a consequence of these features MSCs are being employed as a means of therapeutic immunomodulation for the treatments of autoimmune diseases, graft versus host disease (GvHD) and allograft rejection. Indeed, initial clinical investigations have reported promising results in the treatment of GvHD, Multiple sclerosis and Crohn’s disease [8–10] and there are currently a large number of safety and efficacy clinical trials ongoing to investigate the use of MSCs as a cellular immunotherapy [11]. The effectiveness of MSC-based immunotherapies has been challenged by recent observations showing that systemically delivered MSCs rapidly undergo apoptosis caused by T cell cytotoxicity and accumulate in the lungs where they undergo apoptosis [12,13].

The basis for the use of MSCs as an immune suppressive therapy derives mostly from the evidence generated *in vitro* where inhibitory effects of MSCs on T-cell proliferation are well established [3,4,14–16]. This property of MSCs is likely to reflect a local *in vivo* function during tissue repair. At the core of this inhibition is the cytoplasmic tryptophan-catabolizing enzyme indoleamine 2,3-dioxygenase (IDO) that is produced by human MSCs in response to inflammation and acts to deplete the essential amino acid tryptophan in the local environment[17]. There are however, a number of fundamental unresolved issues regarding the effects of MSCs on immune cell processes, not least the observation that mouse MSCs do not produce IDO but rather inhibit T cell proliferation by Nitric oxide [18,19].

This apparent lack of a common mechanism has hampered progress in this area. We describe here experiments that identify a common downstream effector mechanism of T cell inhibition in both human and mouse MSCs as Endoplasmic Reticulum (ER) stress. In human T cells this inhibition is mediated by IDO depletion of tryptophan acting in a quantal manner to produce an “all-or-nothing” switch at tryptophan concentrations below fluctuations in physiological levels. In mouse cells there is already considerable evidence that NOS impacts upon ER stress and thus this is likely to underpin the local effects of MSCs on T cells and establishes the mouse as an appropriate model to study MSC-T cell interactions.

## Results

### Human dpMSC-mediated inhibition of T-cell proliferation involves a near-binary response to tryptophan starvation

**Fig 1:**
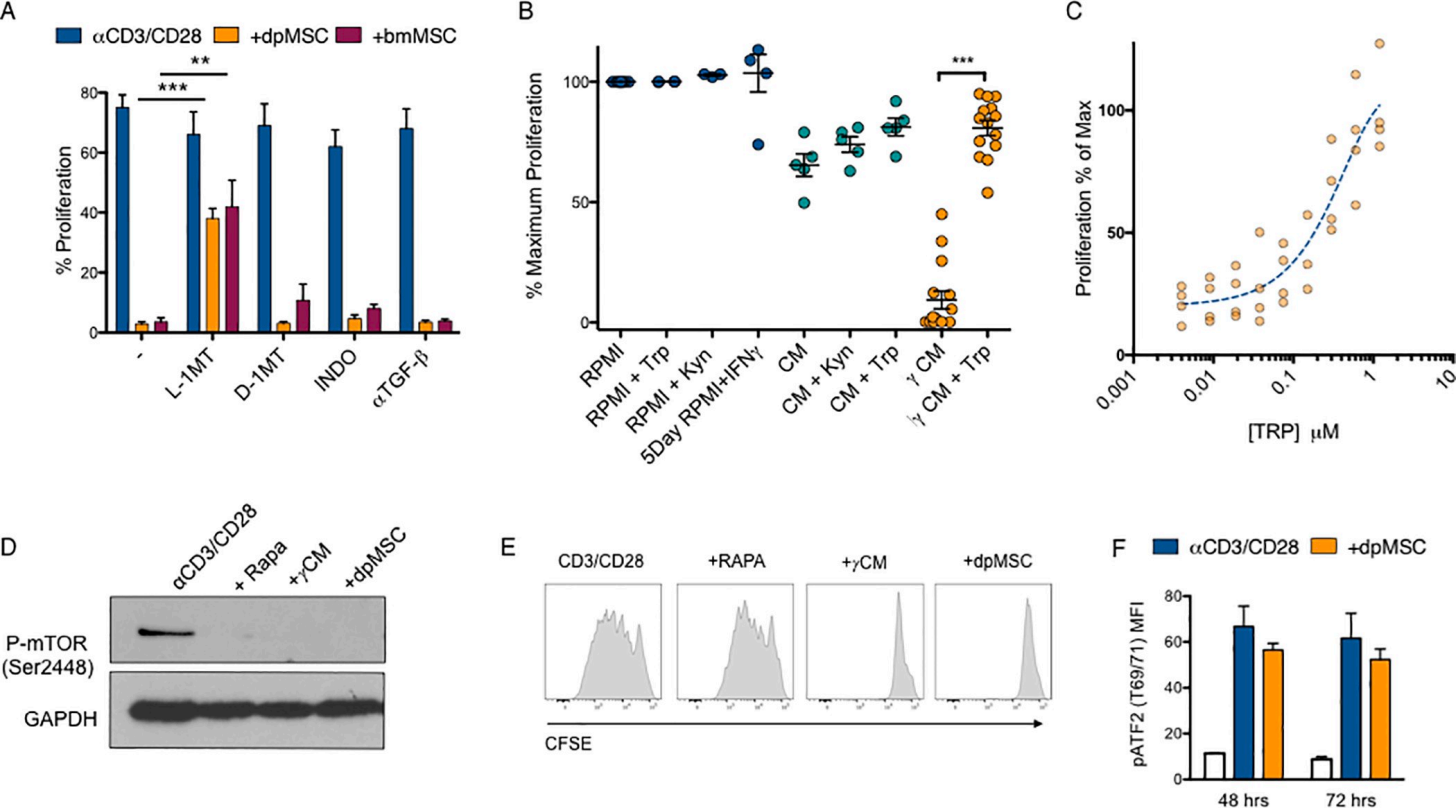
Tryptophan starvation inhibits T-cell proliferation through activation of GCN2 and induction of ER-stress. **(A)** Proliferation of αCD3/CD28-activated CFSE labelled PBMC co-cultured with either bmMSC or dpMSC in the presence of L-1-MT, D-1-MT, indomethacin, or TGF-β neutralizing antibody (n = 6). (**B)** Proliferation of αCD3/CD28-activated CFSE labelled CD4^+^ T-cells cultured in 100% non-activated dpMSC conditioned medium (CM) or IFN-**γ** activated dpMSC conditioned medium (γCM), supplemented with Kynurenine (Kyn) or Tryptophan (Trp) (n = <13). (**C**) αCD3/CD28-activated CD4^+^ T-cells cultured in 100% γCM supplemented with increasing concentrations of Tryptophan (Trp). Proliferation was determined by CFSE dilution (n = 4). (**D**) Representative immunoblot of phopsho-mTOR (pmTOR) in αCD3/CD28-activated CD4^+^ T-cells (72 hours) cultured with Rapamycin (Rapa), Tryptophan (Trp) free conditions or in the presence of dpMSC. (**E)** Representative histograms showing CFSE proliferation of αCD3/CD28-activated CD4^+^ T-cells (72 hours) cultured with Rapamycin, conditioned medium γCM or with dpMSC. Data are pooled from at least two independent experiments. **(F)** Flow cytometry analysis of phospho-ATF2 (MFI) in αCD3/CD28-activated CD4^+^ T cells cultured for 48 and 72 hours in the presence or not of dpMSC. (White) = non-activated T cells; (Blue) = αCD3/CD28; (Orange) = dpMSC.

Inhibition of T-cell proliferation is widely reported in the literature as a feature of cells with defined *in vitro* characteristics of mesenchymal stem cells (MSCs), (expression of markers and induced tri-lineage differentiation), regardless of tissue of origin [20] [21]. Dental pulp (mesenchymal) stem cells (dpMSCs) exhibit qualitatively similar effects on T cell proliferation as bone marrow mesenchymal stem cells (bmMSCs) but because of their accessibility, comparable populations of dpMSCs from humans and mice can be obtained and studied [22,23]. In corroboration with published findings we found that the inhibition of proliferation of αCD3/CD28 activated CD4^+^ T-cells by both dpMSCs and bmMSCs could be partially reversed through the addition of the IDO inhibitor L-1MT, but not D-1MT (**Fig 1A**). The effects could not be reversed by inhibitors of other proposed suppressive mechanisms of MSC-mediated immune suppression including TGF-ß neutralising antibodies, or PGE-2 using the COX2 inhibitor indomethacin **(Fig 1A)**. Having confirmed the importance of IDO in MSC mediated suppression of CD4^+^ T-cells with both dpMSC and bmMSC we wanted to address the precise mechanism of action of IDO in this context. IDO has been proposed to function by a combination of tryptophan starvation and/or the generation of immunologically active tryptophan catabolites collectively called kynurenines. In order to distinguish between these two mechanisms, we were able to utilize the tight regulatory control of IDO expression in MSC by IFNγ [24] and analyse the ability of T cells to proliferate in supernatants from IFN-γ-activated IDO^+^ dpMSCs versus non-activated IDO^-^ dpMSC (CM) supernatants (**Fig 1B**). Our results showed that αCD3/CD28-activated CD4^+^ T cells were unable to proliferate when cultured in 100% conditioned media from IFNγ-licensed MSCs (γCM), but were able to proliferate in 100% from unlicensed MSCs (CM). The dependence of this suppression on tryptophan starvation could be demonstrated by the fact that addition of Tryptophan (Trp) could almost fully rescue T-cell proliferation, in IFNγ-licensed MSC CM. Conversely, the addition of the primary tryptophan catabolite Kynurenine (Kyn) to either culture medium (RPMI) or unlicensed conditioned medium had no effect on T-cell proliferation demonstrating that suppression of CD4 proliferation by IDO in this context is entirely tryptophan dependent (**Fig 1B**). Titration of Tryptophan back into IFNγ-activated IDO^+^ dpMSC CM revealed that the T-cell responsiveness to amino acid starvation occurred over a very narrow window of tryptophan concentrations exhibiting a near binary response to amino acid starvation (dynamic range spanning only a 10 fold change in concentration) **(Fig 1C).**

To identify the cellular signalling pathways responsible for the arrest in T cell proliferation we assessed the impact on known amino acid sensing signalling pathways mTOR and ATF2 [25–27] (**Fig 1D and 1F**). We were able to demonstrate that IDO-mediated tryptophan starvation induced with either conditioned media from IFNγ-licensed MSCs (γCM) or in the presence of MSCs (dpMSC) inhibited mTOR phosphorylation to the same extent as Rapamycin (Rapa) (**Fig 1D**). However, mTOR signalling alone was not able to account for the inhibition of T-cell proliferation mediated via either conditioned media from IFNγ-licensed MSCs (γCM) or in the presence of MSCs (dpMSC), since inhibition of mTOR signalling using Rapamycin had no effect on T-cell proliferation (**Fig 1E**). Similarly, co-culture of CD4^+^ T cells with dpMSCs did not activate the alternate ATF2 amino acid starvation-sensing pathway, thus excluding its contribution to the arrest in T cell proliferation [27] (**Fig 1F).**

### Amino acid starvation by dpMSCs inhibits T cell proliferation through ER stress induction

**Fig 2:**
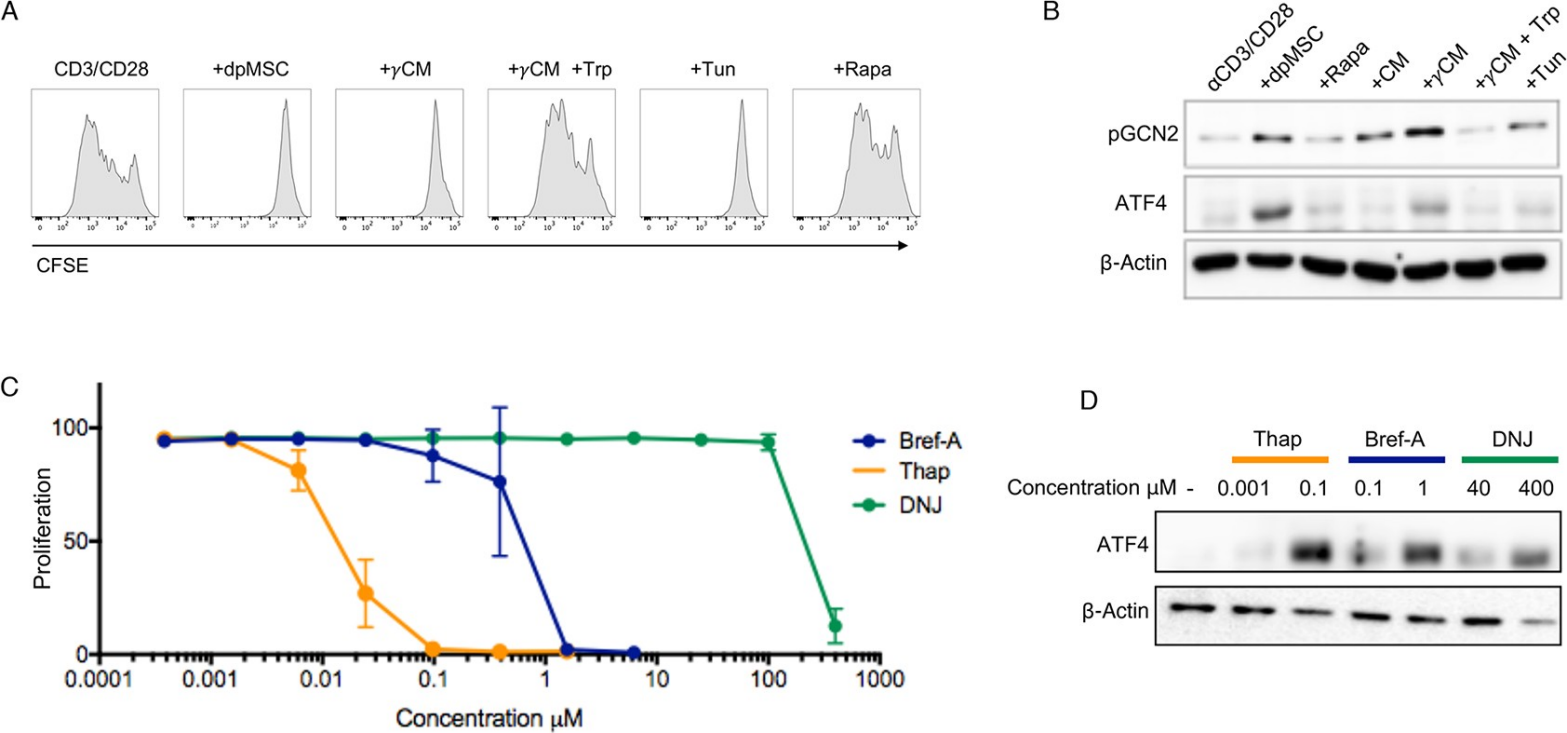
Tryptophan starvation inhibits T-cell proliferation through activation of GCN2 and induction of the cellular stress response. (**A**) Representative histograms showing the proliferation of αCD3/CD28-activated CFSE-labelled CD4^+^ T-cells co-cultured with dpMSC, conditioning medium from IFNγ licenced IDO^+^ dpMSC cultures (γCM) with or without Tryptophan (Trp), Rapamycin (Rapa), or in the presence of the ER-stress inducer Tunicamycin (Tun). Data are representative of at least two independent experiments. (**B**) Representative immunoblot showing the expression of pGCN2 and ATF4 in αCD3/CD28-activated CD4^+^ T-cells upon direct co-culture with dpMSC, Rapamycin (Rapa), CM, γCM in the presence or absence of Tryptophan (Trp) or in the presence of the ER-stress inducer Tunicamycin (Tun). (**C**) Proliferation of αCD3/CD28-activated CD4^+^ T-cells, as measured by CFSE dilution, in the presence of varying concentrations of three ER-stress inducers (Bref-A, Thap, DNJ). Data are pooled from at least two independent experiments. (**D**) Representative immunoblot showing the expression of ATF4 in αCD3/CD28-activated CD4^+^ T-cells in response to ER-stress inducing compounds at concentrations that straddle their inhibition of T-cell proliferation.

Having shown that the ATF2 amino acid starvation-sensing pathway was not involved in the inhibition of T cell proliferation, we postulated that additional amino acid sensing pathways could be implicated in this process. The GCN2 amino acid sensing pathway has been demonstrated to be non-redundant in controlling the proliferation of T-cells in response to amino acid starvation in the mouse [28–30]. To date however, the importance of this pathway in MSC-mediated amino acid starvation response in human T cells has not yet be reported. To address this, we cultured aCD3/CD28-activated CD4^+^ T-cells in different conditions (**Fig 2A**). αCD3/CD28 stimulation was selected because it mimics the classic two signal models for T cell activation, namely ligation by the TCR and the MHC-P complex and by the CD28 of the CD80 and CD86. Other combination of antibodies can lead to T cell stimulation but the outcome can be different, e.g. induction of regulatory T cells. As shown before, T cell proliferation mediated by αCD3/CD28 was inhibited by dpMSC and conditioning medium from IFNγ licenced IDO^+^ dpMSC cultures (γCM). The effect of γCM was reversed by the addition of Tryptophan, while Rapamycin did not have an effect (**Fig 2A**). T cells were then stimulated for 4 hours only under the same conditions reported in Fig 2A and assayed the phosphorylation of GCN2, and the expression of the downstream ER-stress associated effector ATF4. We found that both pGCN2 and ATF4 levels increased in CD4^+^ T-cells conditioned media from IFNγ-activated dpMSCs, and that this could be fully reversed by the addition of Tryptophan **(Fig 2B).** This result was confirmed when T cells were co-cultured with dpMSC, while the absence of any effect of Rapamycin on the ER-stress signalling signature of amino acid depravation confirmed the lack of any T-cell inhibition (**Fig 2B**).

Whilst GCN2 has been shown to be an important component of the amino acid starvation response in mice [30], it is also a more generalizable feature of the cellular stress response [31], and its phosphorylation in response to amino acid starvation coupled with the increase in downstream ER-stress associated effector transcription factor ATF4 prompted us to ask whether chemical induction of ER-stress could mimic amino acid starvation-induced inhibition of T cell proliferation. To this end, we cultured αCD3/CD28-activated CD4^+^ T-cells in the presence of the ER-stress inducer Tunicamycin (Tun) and showed that this was able to replicate not only the ER-stress signalling signature of amino acid depravation, but also the inhibitory effects on T-cell proliferation to the same extent as direct co-culture with dpMSC or culture in γCM (**Fig 2A and 2B)**.

To further confirm that ER stress may be causative in the inhibition of T-cell proliferation, we assessed the effects of three mechanistically distinct ER-stress inducing compounds; Thapsagargagin (TG)–an inhibitor of the sarco/endoplasmic reticulum Ca2+ ATPase, the ER protein transport inhibitor Brefeldin-A (BrefA), and the ER glucosidase inhibitor *N*-butyl-deoxynojirimycin (NB-DNJ) [32,33]. In each case induction of ER-stress, as determined by ATF4 upregulation, correlated with inhibition of T-cell proliferation **(Fig 2C and 2D)**.

### ER-stress inhibits T-cell activation

**Fig 3:**
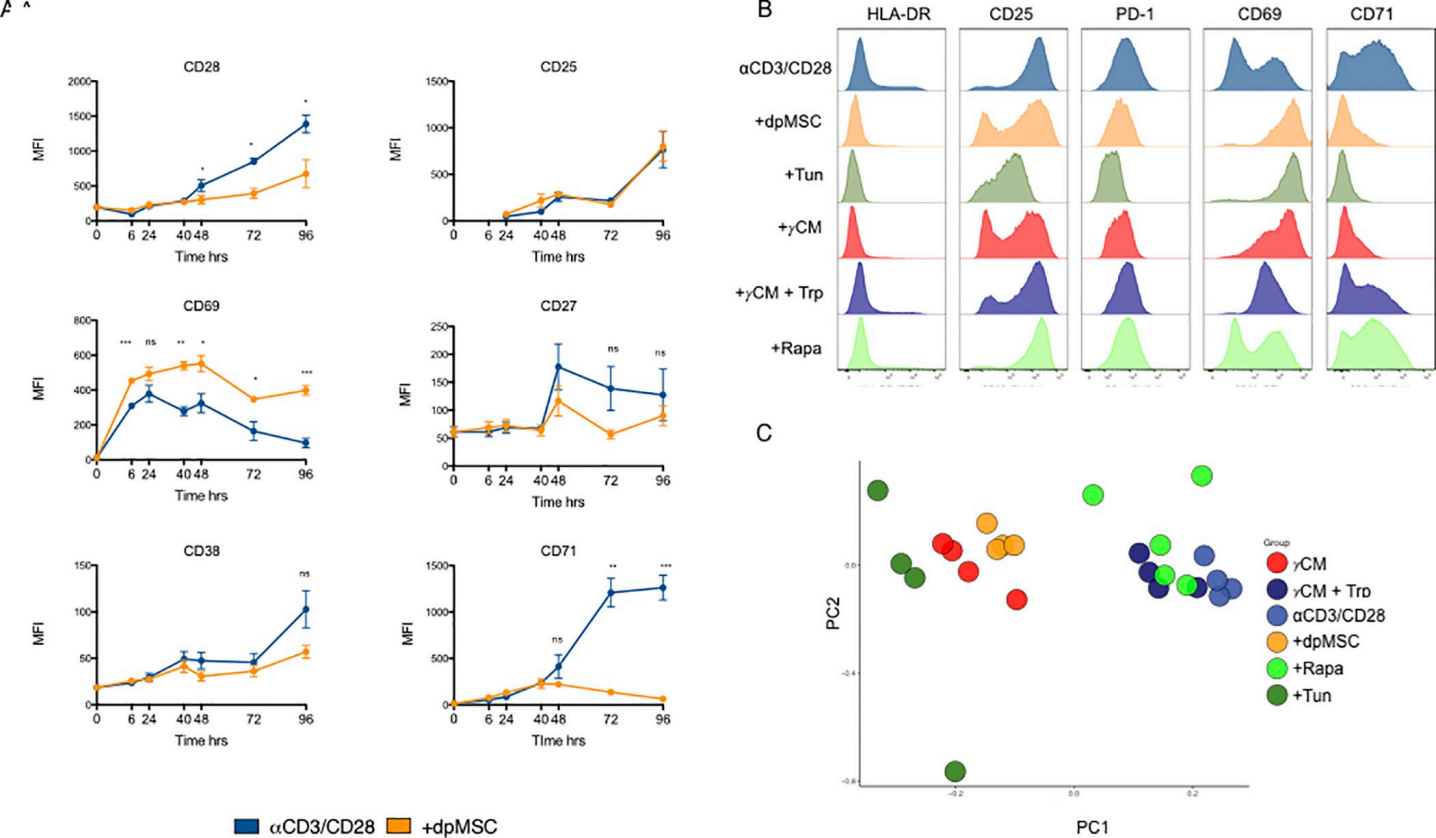
Cellular stress inhibits T-cell activation. **(A)** Flow cytometry analysis of T-cell activation marker expression (MFI) in αCD3/CD28-activated CD4^+^ T-cells cultured alone or in the presence of dpMSC (n = 4). **(B)** Representative histograms showing T-cell activation marker expression at 72 hours in αCD3/CD28 activated CD4^+^ T-cells cultured alone, in the presence of dpMSC, Tunicamycin (Tun) 100% IFNγ-activated dpMSC CM or in IFNγ activated dpMSC supplemented with tryptophan and rapamycin (Rapa). (**C**) Principal component analysis of activated cell subset phenotypes in CD4^+^ T cells cultured under the conditions indicated.

To assess the ability of dpMSCs to influence other T cell functions, we asked to what extent dpMSCs were able to inhibit activation as determined by expression levels of several T cell activation markers. To this end, we co-cultured αCD3/CD28-activated CD4^+^ T cells with dpMSCs and assessed their activation phenotype over the course of 5 days. Co-culture with dpMSC led to a reduction in T-cell activation as determined by a reduction in expression of the activation markers, CD38, CD27, CD28 and CD71 but not CD25 or CD69 (**Fig 3A**). Interestingly, CD69 expression was increased suggesting an imprinting of a tissue resident phenotype on cells in the context of co-culture conditions or an arrest in T-cell activation dynamics **(Fig 3A)**.

To assess the contribution of amino acid starvation and ER stress induction to this inhibition of T-cell activation, we cultured CD4^+^ T cells in the presence of dpMSC, IFNγ-licensed IDO^+^ dpMSC CM +/- Tryptophan, Rapamycin, or in the presence of Tunicamycin (Tun) for 72 hours (**Fig 3B**). T-cells cultured in IDO^+^ dpMSC conditioned medium displayed identical CD69 and CD71 activation marker expression to CD4^+^ T cells in direct dpMSC co-cultures and this effect could be partially reversed by the addition of exogenous Tryptophan (Trp). Similarly, the altered activation phenotype could be directly reproduced by chemical induction of ER-stress using Tunicamycin, (Tun). Principal component analysis of the expression levels of all T-cell activation markers revealed two discrete clusters that corresponded with activation of the ER-stress pathway in CD4^+^ T cells **(Fig 3C)**. Together these findings highlight ER-stress as a potent modulator of T cell activation induced by MSCs.

### ER-stress is a conserved mechanism used by MSCs to modulate T cell proliferation

**Fig 4:**
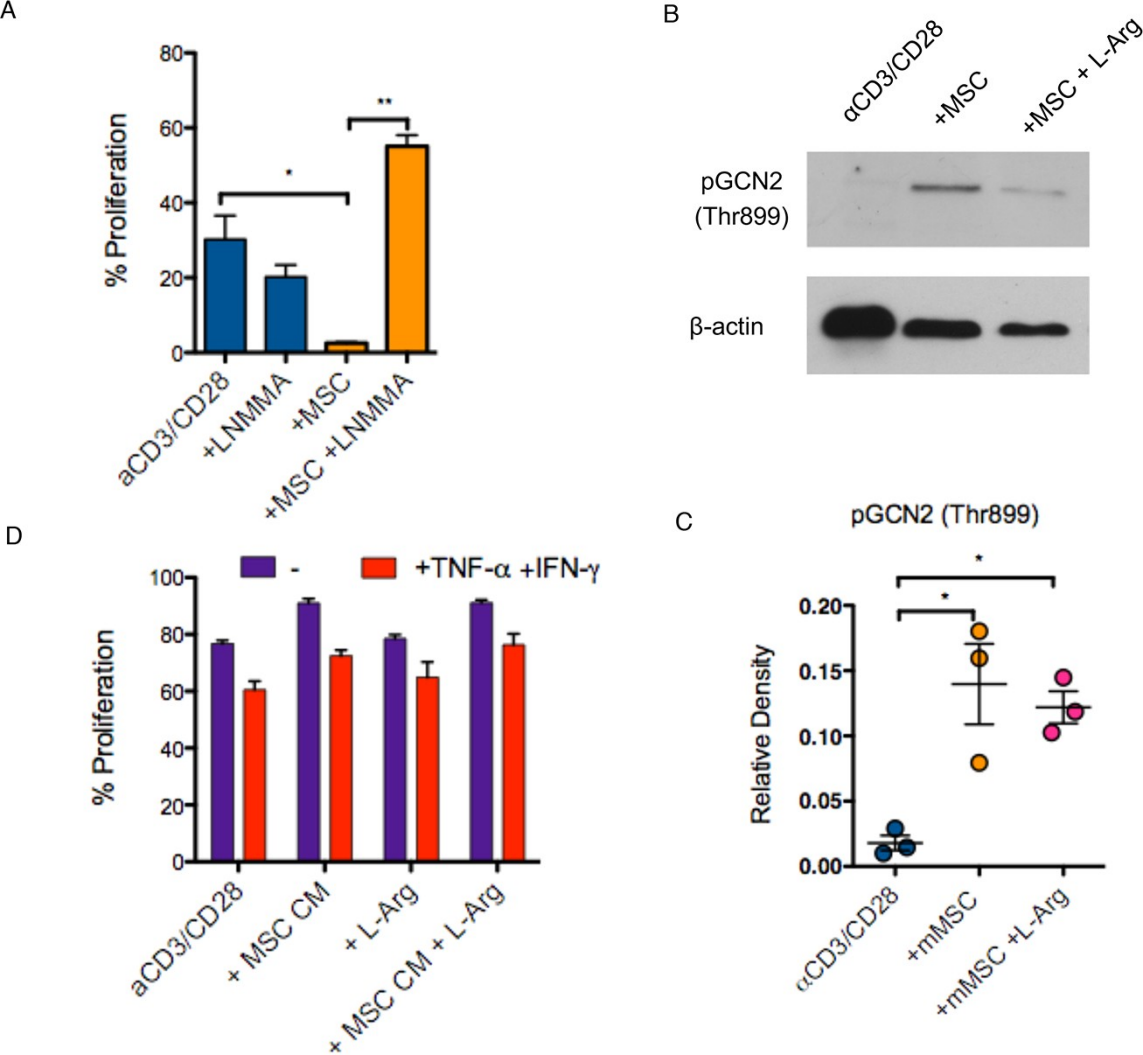
Mouse MSCs inhibit T-cell proliferation via NO production and induction of cellular stress response. **(A)** Proliferation of αCD3/CD28-activated CFSE-labelled CD4^+^ T-cells cultured in direct contact with mMSC ± iNOS inhibitor LNMMA (n = 4). **(B)** Representative immunoblot and **(C)** quantification of GCN2 phosphorylation (pGCN2) in αCD3/CD28-activated CD4^+^ T-cells co-cultured with mMSC or mMSC in the presence of L-Arg. **(D)** Proliferation of αCD3/CD28-activated CFSE-labelled CD4^+^ T-cells cultured in 100% non-activated or IFN-γ/TNF-α activated mMSC-conditioned medium.

Previous reports have indicated that murine MSCs differ from human MSCs in the mechanism of inhibition of T-cell proliferation, being dependent on iNOS as opposed to IDO [19]. To confirm this, we inhibited iNOS using LNMMA during mouse dpMSC and murine CD4^+^ T-cell co-cultures and observed restoration of T-cell proliferation induced by αCD3/CD28 **(Fig 4A)**. We considered two hypotheses to explain the inhibition of T-cell by iNOS. First, it is possible that iNOS has the potential to inhibit T-cell proliferation through the depletion of its target substrate L-Arginine. In principle, arginine depletion could induce ER-stress in a manner directly analogous to IDO-mediated tryptophan starvation. Alternatively, we considered that iNOS may act through the production of nitric oxide itself, which again has the potential to induce ER-stress via promoting oxidative stress [34,35]. Co-culture of mouse dpMSCs with autologous αCD3/CD28 activated CD4^+^ T-cells led to the phosphorylation of the ER-stress protein GCN2 (**Fig 4B and 4C**). This effect could not be reversed through the addition of an excess of L-Arg, indicating that arginine-depletion is not responsible for the induction of ER stress. Indeed, the suppressive properties of mouse dpMSCs could not be reproduced using conditioned media from activated mouse dpMSCs cultures as would be expected if arginine depletion were the mechanism of suppression (**Fig 4D**). Thus, although mouse MSCs induce ER stress in T-cells, they do so via an amino acid depletion-independent mechanism that most likely involves the production of NO. These findings reconcile the apparently different mechanisms used by mouse and human MSCs. In both cases the same downstream mechanism is employed, namely ER stress but in each case, this is achieved by different upstream effectors.

## Discussion

MSCs are the focus of clinical research studies due to their potential use for cellular immunotherapy [9]. This interest has been fuelled over the past years by multiple *in vitro* studies showing strong immunomodulatory properties, with the principal effector being the potent inhibition of T cell function [36]. The findings of these *in vitro* studies in human cells have been the identification of enzyme-mediated tryptophan depletion by MSCs as the main mediator of inhibition of T-cell proliferation. When mouse-derived MSCs were used in these assays, amino acid depletion was not observed and rather the production of nitric oxide (NO) by mouse cells has been implicated as the mechanisms of action [18].

In order to provide a better appreciation of the importance of MSC-mediated modulation of T cell response *in vitro* and also inform the use of MSCs in a therapeutic setting, we set out to understand more of the interactions between human and mouse MSCs and T cells. We focused on cells from tooth pulp (dpMSCs) since this allowed us to directly isolate and culture fresh cells from both humans and mice.

We found that a common downstream mechanism exists in both mouse and human dpMSCs, whereby ER stress is induced in T-cells and this prevents T cell activation. In human dpMSCs we confirmed that production of IDO and subsequent depletion of tryptophan inhibits T cell proliferation over a narrow range of concentrations producing a near-binary response between 0.1–1.0 γM tryptophan. Although we cannot rule out hidden effects on T cell subtypes, this narrow concentration range further supports the effects on T cells are likely to be restricted to the immediate, local environment rather than systemic.

In mouse dpMSCs however we identified iNOS as being required for inhibition of T cell proliferation, exerting its effects not through amino acid depletion but via production of NO. A common feature of both amino acid starvation (human) and NO production (mouse) as means of immune modulation is their extremely limited range of action, NO having a half-life of <3 seconds and amino acid starvation, as we have shown, relying on total deprivation; an effect which would only be achievable locally[37]. Both these mechanisms appear tailored to extremely localized immunosuppression that is consistent with the proposed role of MSCs as tissue resident mediators of innate tolerance. In keeping with this hypothesis is the observation that the direct local immunosuppressive effects of stromal cells are sufficient to inhibit the anti-tumour immune response *in vivo* [38]. In addition, although no direct implication of stromal cells, IDO has been shown to play an important role in immune evasion by tumours [39,40]. Beyond these mechanistic considerations there exists two distinct lines of evidence directly linking the immunosuppressive properties of MSCs with their biological function *in situ*. First, there are circumstances whereby the tolerogenic effects of MSCs could be deleterious e.g. during infection where immunosuppression by MSCs would favour the progression of the infection within a tissue, and consequently it would be essential to adequately control these effects *in vivo*. However, the finding that TLR ligation inhibits the immunosuppressive properties of MSCs [41,42] provides evidence that immunosuppression by MSCs is only deployed in instances of sterile inflammation e.g. following the immunological resolution of infection. Secondly, it has been demonstrated that inflammation can directly inhibit MSC differentiation [43], consistent with the idea that MSC differentiation and ultimately tissue repair and regeneration requires resolution of the immune response associated with tissue damage.

The recent observations highlighting the role of MSC apoptosis as a primary mechanism of immune modulation following systemic injection of MSCs emphasises the need for a better understanding of the natural *in vivo* local mechanisms [13,44]. We therefore propose a model whereby the local control of the immune response within the tissue is both essential to protect the tissue resident stem cell population and important in the timely resolution of the immune response, thus allowing MSCs to initiate repair programs following tissue damage in a coordinated manner, balancing the competing imperatives of tissue repair and elimination of infection.

## Material and methods

### Ethical statement

Deciduous teeth were collected from patients having given informed consent and ethical approved was given by the NHS Research Ethics Committee. All methods were carried out in accordance with guidelines and regulations as licensed by the HTA at King’s College.

Animal experiments were carried out in accordance to approved Home Office regulations.

### Cell isolation and separation

Pulp tissue was extracted and digested with 200u/ml collagenase II (Worthington) at 37°C for 1 hour. Bone marrow mononuclear cell fractions were purchased from Lonza, (n = 9). Both bone marrow MSCs (bmMSCs) and dental pulp MSCs (dpMSCs) were cultured in 10% DMEM at 37°c 5%CO_2._ Peripheral blood mononuclear cells (PBMCs) from healthy donors were obtained from anonymized leukocyte cones supplied by the National Blood Transfusion Service (NHS Blood and Transplantation, Tooting, London, UK). PBMCs were isolated by Lymphocyte (PAA, Austria) density gradient. RosetteSep Human CD4^+^ T cell enrichment cocktail (STEM CELL, Cambridge, UK) was used to obtain purified CD4^+^ T cells. The purity of CD4^+^ T cells was between 95–99%. Mouse bmMSCs were isolated from hind limb tibia of C57BL/6j mice and cultured in alphaMEM + 20% FCS at 37°c 5%CO_2_. Mouse CD4^+^ T-cells cells were isolated from solenocytes by negative depletion using mouse CD4^+^ T-cell isolation kit (Miltenyi). C57BL/6j were maintained under pathogen-specific sterile conditions in the Biological Services Unit at King’s College London. All procedures were performed in accordance with institutional guidelines and the Home Office Animals Scientific Procedures Act (1986).

### Proliferation assays

bmMSCs and dpMSCs were co-cultured for 5 days with 1×10^5^ CFSE labelled PBMCs or CD4^+^ T cells in RPMI 10% FCS. CD4^+^ T cells were activated using αCD3/CD28 micro-beads (Invitrogen, UK) at a concentration of 3μl/10^6^ T cells. Cell proliferation was determined by monitoring CFSE dilution.

### Conditioned medium experiments

dpMSCs were cultured in the same format as in previous direct co-culture conditions i.e. in the same density and volume of culture media. dpMSCs were activated with 1000iu/ml recombinant hIFN-γ (R&D, Abingdon,UK) for 5 days. CFSE labelled PBMCs were washed twice in PBS to remove any residual tryptophan following CFSE staining and cultured in 100% dpMSC conditioned media and activated with αCD3/αCD28 micro-beads (Invitrogen, UK) at a concentration of 3μl/10^6^ PBMCs. Cell proliferation was determined by CFSE dilution.

### Flow cytometry

Cell surface staining was performed in PBS 2% FCS 0.5mM EDTA for 20 minutes at 4^o^c. bmMSCs and dpMSCs were stained with the following markers: CD146 FITC, CD271 FITC, STRO1 PE, CD90 PE (all Abcam, Cambridge, UK) and CD45 FITC (eBioscience, Hatfield, UK). CD4^+^ T cells were analyzed for the following markers: CD71, CD69, CD27, CD38, CD25 and CD28 (all eBioscience, Hatfield, UK).

Cells were acquired on 4-laser BD LSR Fortessa. (S1 Fig).

### Western blotting

CD4^+^ T cells were lysed in 50ul of lysis buffer (4.8% SDS, 8% sucrose, 2M urea) containing protease inhibitor cocktail (Calbiochem), for 30 minutes on ice and centrifuged for 15 minutes at 15,000 rpm. Protein concentrations were determined by Quick Start Bradford assay kit (Bio-Rad), according to the manufacturer's instructions. Protein lysates were denatured at 95°C for 5 minutes. Protein samples (25μg) were separated on 10% or 12% sodium dodecyl sulfate-polyacrylamide gels and transferred onto polyvinylidene difluoride (PVDF) membranes (Millipore) and probed using the following antibodies: pGCN2, ATF4, GAPDH and ß-actin (Cell Signalling, Danvers, MA, USA). Detection of the immunoreactivity bands was performed with the ECL Western Blotting Substrate (Bio-Rad), and chemiluminescence was detected with the ImageQuant imaging system using anti-rabbit or anti-mouse HRP-linked antibody (eBioscience).

### Statistics

Statistical analysis was performed using GraphPad Prism V7.0 Statistical significance between two experimental groups was performed using two tailed students T-test. Analysis of more than two groups was performed using ANOVA. P-values denoted as follows: *P < 0.05, **P < 0.01, and ***P < 0.001.

## Supporting information

S1 Fig **(A)** Flow cytometry analysis representative of multiple donors of cultured bmMSCs and dpMSCs. Solid grey peak represents isotype matched control antibody; solid black line peak represents specific staining. **(B)** bmMSC and dpMSC cultured in chondrogenic (Ch), osteogenic (Os) and adipogenic (Ad) differentiation condition. (i) chondrogenic differentiation of micro-mass cultures as determined by Alcian blue staining; (ii) osteogenic differentiation as determined by alkaline phosphatase activity and mineralization as determined by alizarin red staining (iii); adipogenic differentiation as determined by Oil Red O staining (iv). **(C)** Colony forming unit fibroblasts (CFU-F) was assessed in bmMSC and SHED at P4 Colonies exceeding fifty cells in number were counted; graph represents average number of colonies/100cells.
(DOCX)
