# Supplementary material for: Mesenchymal stem cells inhibit T-cell function through conserved induction of cellular stress
Source: PLoS One. 2019 Mar 14;14(3):e0213170. doi: 10.1371/journal.pone.0213170 (PMC6417714; doi:10.1371/journal.pone.0213170)
Supplement: S1 Fig — (A) Flow cytometry analysis representative of multiple donors of cultured bmMSCs and dpMSCs. Solid grey peak represents isotype matched control antibody; solid black line peak represents specific staining. (B) bmMSC and dpMSC cultured in chondrogenic (Ch), osteogenic (Os) and adipogenic (Ad) differentiation condition. (i) chondrogenic differentiation of micro-mass cultures as determined by Alcian blue staining; (ii) osteogenic differentiation as determined by alkaline phosphatase activity and mineralization as determined by alizarin red staining (iii); adipogenic differentiation as determined by Oil Red O staining (iv). (C) Colony forming unit fibroblasts (CFU-F) was assessed in bmMSC and SHED at P4 Colonies exceeding fifty cells in number were counted; graph represents average number of colonies/100cells. (DOCX) [file pone.0213170.s001.docx]

**Supplementary data**

**Mesenchymal stem cells inhibit T-cell function through conserved induction of cellular stress**

Adam G. Laing ^1,2*^, Giorgia Fanelli^1*^, Andrei Ramirez-Valdez ^2,3^, Robert I. Lechler^1^, Adrian Hayday^3,4^, Giovanna Lombardi^1*^ and Paul T. Sharpe^1,2*^

**Figure S1**

**Figure S1. (A)** Flow cytometry analysis representative of multiple donors of cultured bmMSCs and dpMSCs. Solid grey peak represents isotype matched control antibody; solid black line peak represents specific staining. **(B)** bmMSC and dpMSC cultured in chondrogenic (Ch), osteogenic (Os) and adipogenic (Ad) differentiation condition. (i) chondrogenic differentiation of micro-mass cultures as determined by Alcian blue staining; (ii) osteogenic differentiation as determined by alkaline phosphatase activity and mineralization as determined by alizarin red staining (iii); adipogenic differentiation as determined by Oil Red O staining (iv). (**C)** Colony forming unit fibroblasts (CFU-F) was assessed in bmMSC and SHED at P4 Colonies exceeding fifty cells in number were counted; graph represents average number of colonies/100cells.
